# Supplementary figures and images for: Bidirectional Control of Absence Seizures by the Basal Ganglia: A Computational Evidence
Source: PLoS Comput Biol. 2014 Mar 13;10(3):e1003495. doi: 10.1371/journal.pcbi.1003495 (PMC3952815; doi:10.1371/journal.pcbi.1003495)

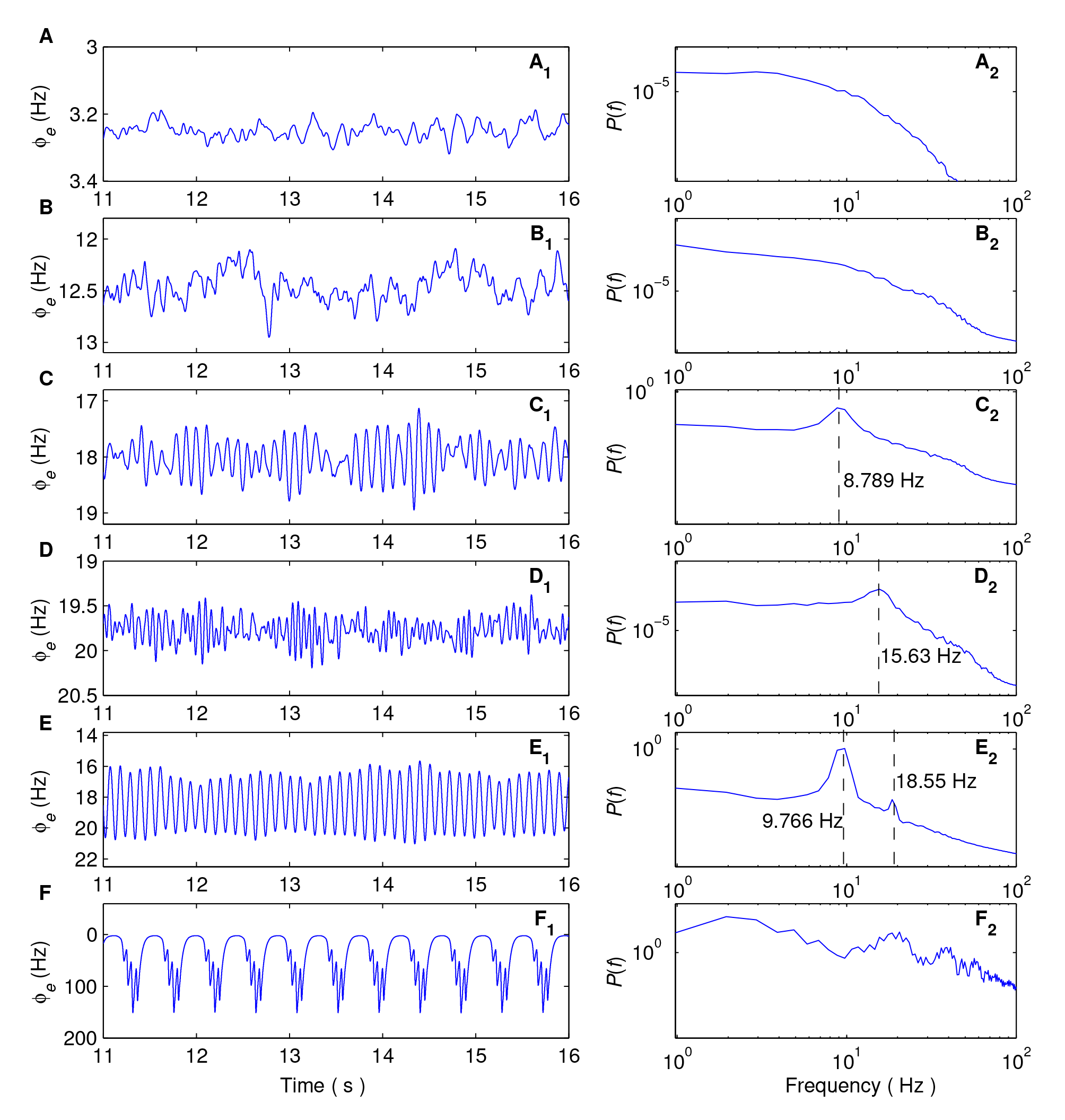

Supplement: Figure S1 — Several other typical time series of (left frames) and their corresponding spectra (right frames) generated by our developed BGCT model. To a certain extent, these model time series are comparable with real physiological EEG signals: eyes-open (A) and (B), alpha rhythm (C), beta rhythm (D), coexistence of alpha and beta rhythms (E), and polyspike and wave (F). Here we adjust the excitatory corticothalamic coupling strength and delay parameter for reproducing these time series. The detailed parameter values used in our simulations are: and (A), and (B), and (C), and (D), and (E), and and (F), respectively. In addition, we also introduce a certain level of gaussian white noise into , with the mean and standard deviation . Note that more types of model time series might be observed by further tuning these critical parameter values. (TIF) [file pcbi.1003495.s001.tif]

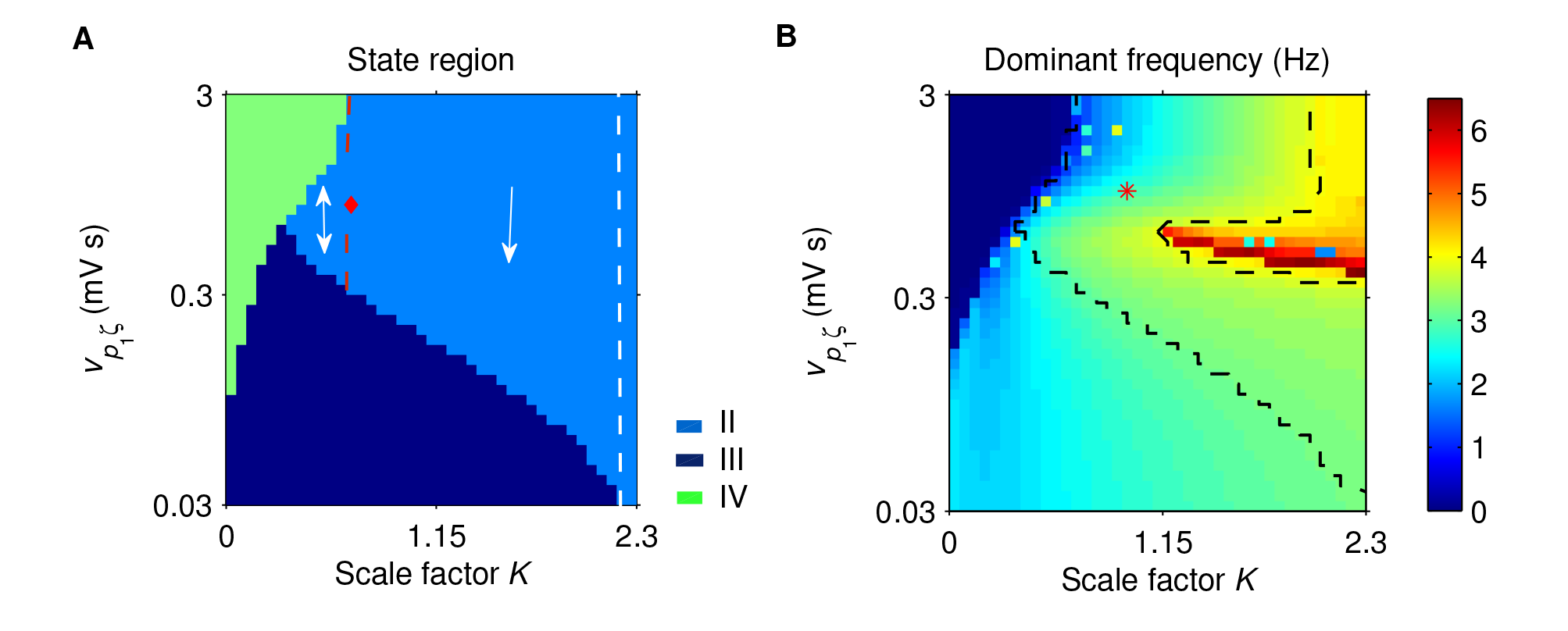

Supplement: Figure S2 — An example representation of the bidirectional control of absence seizures by the basal ganglia in a relatively larger scale factor interval. The state analysis (A) and frequency analysis (B) in the () panel, with the inhibitory coupling strength . Here is the scale factor, and is the excitatory coupling strength of the STN-SNr pathway used to control the activation level of SNr neurons. Similar to the results in Figs. 5A and 6A, only three dynamical state regions are observed in the phase diagram (A): the SWD oscillation region (II), the simple oscillation region (III) and the low firing region (IV). In (A), the region marked by red diamond denotes the whole suppression regions of SWDs, the white dashed line represents the boundary of suppression region, and the red dashed line stands for the demarcation between the bidirectional (double arrow) and unidirectional (single arrow) suppression regions. In (B), the red asterisk region surrounded by dashed lines denotes the typical 2–4 Hz SWD oscillation region. Compared to the results shown in Figs. 5 and 6, here we consider a relatively larger scale factor interval from 0 to 2.3. (TIF) [file pcbi.1003495.s002.tif]

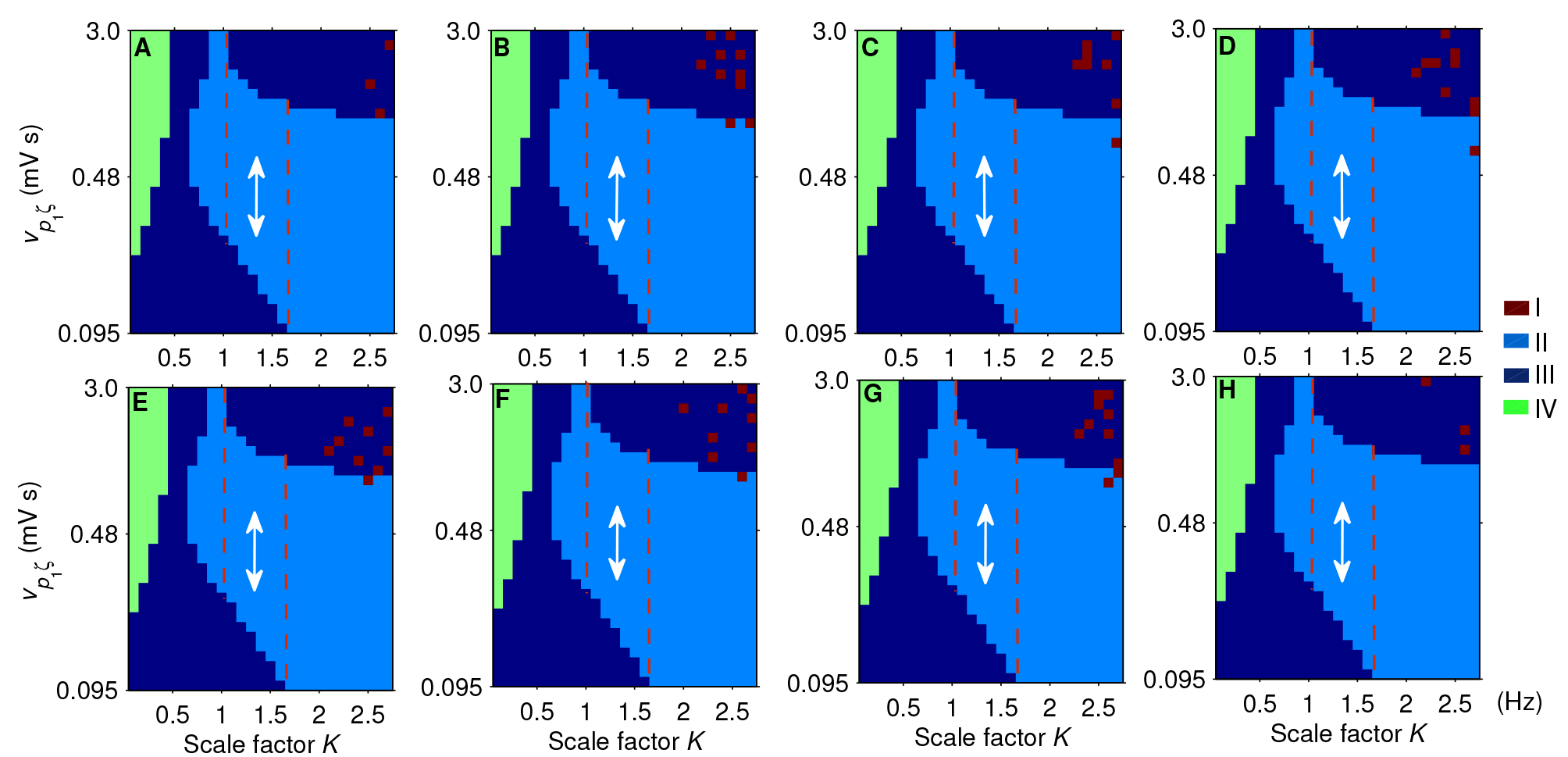

Supplement: Figure S3 — A series of two-dimensional state analysis in the ( ) panel for the modified model. In (A)–(H), we use the same group of parameter values but different random initial conditions for simulations. Similar to previous results, four different dynamical states are observed: the saturation state (I), the SWD oscillation state (II), the simple oscillation state (III) and the low firing state (IV). In each subfigure, the region between two red dashed lines denotes the main bidirectional suppression region of SWDs, where the double arrow represents that both increasing and decreasing can inhibit the generation of SWDs. The results given in (A)–(H) indicate that the modified model shows bistability (the simple oscillation state or the saturation state) in the large and strong region, and the final dynamics of the modified model significantly depend on the initial conditions. Note that in all simulations, we set , , , , and . (TIF) [file pcbi.1003495.s003.tif]
